# Regulation of Energy Metabolism by Receptor Tyrosine Kinase Ligands

**DOI:** 10.3389/fphys.2020.00354

**Published:** 2020-04-21

**Authors:** Meng Zhao, Yunshin Jung, Zewen Jiang, Katrin J. Svensson

**Affiliations:** ^1^Department of Pathology, Stanford University, Stanford, CA, United States; ^2^Stanford Diabetes Research Center, Stanford, CA, United States

**Keywords:** receptor tyrosine kinases, metabolism, glucose, lipids, signaling

## Abstract

Metabolic diseases, such as diabetes, obesity, and fatty liver disease, have now reached epidemic proportions. Receptor tyrosine kinases (RTKs) are a family of cell surface receptors responding to growth factors, hormones, and cytokines to mediate a diverse set of fundamental cellular and metabolic signaling pathways. These ligands signal by endocrine, paracrine, or autocrine means in peripheral organs and in the central nervous system to control cellular and tissue-specific metabolic processes. Interestingly, the expression of many RTKs and their ligands are controlled by changes in metabolic demand, for example, during starvation, feeding, or obesity. In addition, studies of RTKs and their ligands in regulating energy homeostasis have revealed unexpected diversity in the mechanisms of action and their specific metabolic functions. Our current understanding of the molecular, biochemical and genetic control of energy homeostasis by the endocrine RTK ligands insulin, FGF21 and FGF19 are now relatively well understood. In addition to these classical endocrine signals, non-endocrine ligands can govern local energy regulation, and the intriguing crosstalk between the RTK family and the TGFβ receptor family demonstrates a signaling network that diversifies metabolic process between tissues. Thus, there is a need to increase our molecular and mechanistic understanding of signal diversification of RTK actions in metabolic disease. Here we review the known and emerging molecular mechanisms of RTK signaling that regulate systemic glucose and lipid metabolism, as well as highlighting unexpected roles of non-classical RTK ligands that crosstalk with other receptor pathways.

## Introduction

The prevalence of obesity and diabetes is a growing health problem with more than a third of the US population now considered obese (Ncd Risk Factor Collaboration [Ncd-RisC], 2016; Zheng et al., 2018). In mammals, energy homeostasis is the balance between energy input and output. The homeostatic control of energy balance is mainly determined by food intake and energy expenditure (Spiegelman and Flier, 2001). Prolonged surplus in energy imbalance leads to weight gain and greatly increases the risk of chronic metabolic disorders such as type 2 diabetes, cardiovascular disease, leading to increased overall mortality (Hruby and Hu, 2015).

Organisms regulate whole-body energy homeostasis through both peripheral and central actions (Friedman, 1995). These cellular signal transduction processes are highly regulated temporal and dynamic events that control basic cellular functions (McKay and Morrison, 2007). Receptor tyrosine kinases (RTKs) are high-affinity cell surface receptors for endocrine or paracrine polypeptide growth factors, hormones, and cytokines and represent a fascinating area of biology. RTKs are responsible for inducing rapid intracellular signaling responses to regulate cell proliferation, survival, motility, metabolism and gene transcription (Schlessinger, 2014). The family consists of 20 identified RTK classes comprising 58 receptor tyrosine kinase proteins. All RTKs share similar overall structural architecture with an extracellular ligand-binding domain, a transmembrane domain, an intracellular regulatory region, a tyrosine kinase domain, and a C-terminal tail (Lemmon and Schlessinger, 2010). Normally, RTKs on the cell surface, whether monomeric or dimeric, are inactive in the absence of a ligand. Upon ligand activation, most RTKs undergo dimerization which juxtaposes the tyrosine kinase domains and facilitates autophosphorylation of the cytoplasmic domain (Hubbard, 2004). While most RTKs consist of a single polypeptide chain, the insulin receptor (IR) and insulin-like growth factor-1 receptor (IGF-1R) are disulfide-linked α2β2 heterodimers. In this case, ligand binding leads to an activation of the pre-existing dimer. The cytoplasmic phosphotyrosine residues, among other residues, serve as dynamic and reversible recruitment sites for adaptor and scaffolding proteins (Sawyer, 1998). Depending on the ligand, the intracellular mediators activated including Src, PLCγ, and PI3K, and docking proteins such as IRS or FRS, determine the downstream effector cascades associated with the activation of the specific receptor (McKay and Morrison, 2007). This leads to the transmission of downstream signals including phosphorylation of kinases in the RAS/MAP and PI3K/AKT pathways, which further contribute to the diversity of cellular responses to a specific ligand (Regad, 2015).

As a ligand of the insulin receptor, insulin is the most well-known endocrine RTK ligand and an anabolic factor that controls whole-body glucose metabolism by increasing glucose uptake in peripheral tissues. Individuals with insufficient production of insulin develop type 1 diabetes, a condition that was fatal before the introduction of purified insulin as a treatment strategy many decades ago. Type 2 diabetes is defined by insulin resistance and compromised insulin secretion. In type 2 diabetic individuals, excess lipid accumulation impairs peripheral insulin signaling in the liver and skeletal muscle which leads to dysregulated cellular lipid and glucose homeostasis and hyperglycemia (Saltiel and Kahn, 2001; Samuel and Shulman, 2016). Insulin has been extensively studied as the first blood glucose-lowering hormone since its discovery nearly 100 years ago (Vecchio et al., 2018). The action of insulin as a receptor tyrosine kinase ligand will therefore not be directly covered in this review, but it exemplifies the powerful actions that a single ligand-receptor interaction can have on whole-body physiology (Vecchio et al., 2018).

In the past 20 years, studies of other RTKs in systemic energy homeostasis have revealed unexpected diversity in RTK regulation of metabolic functions and their mechanisms of actions. For example, endocrine or paracrine ligands of the fibroblast growth factor family, namely FGF1, FGF15/19, and FGF21, have shown to be potent regulators of glucose and lipid metabolism by acting on peripheral organs or in the central nervous system (Markan and Potthoff, 2016). Although less characterized in the context of energy metabolism, several other families of RTKs have demonstrated regulatory functions on cellular and physiological glucose and lipid homeostasis, including platelet-derived growth factor receptors (PDGFRs), hepatocyte growth factor receptors (HGFRs), and the RET receptor.

**FIGURE 1:**
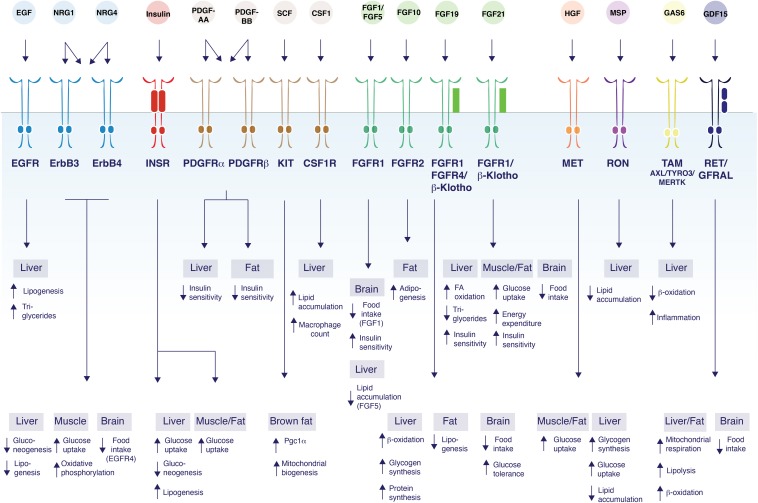
Control of glucose and lipid metabolism by RTK ligands. The schematic figure shows the diversity of functions mediated by RTK ligands and their respective receptors and their tissues of action. EGF binds EGFR to induce lipogenesis in the liver, and increase TG secretion (Scheving et al., 2014). NRG1 acts on ErbB3 and/or ErbB4 to inhibit gluconeogenesis in the liver (Ennequin et al., 2015; Zhang et al., 2018), and to increase glucose uptake and oxidative phosphorylation in myotubes (Canto et al., 2004, 2007; Suárez et al., 2001). NRG1 also decrease food intake by acting on ErbB4 in the brain (Ennequin et al., 2015; Zhang et al., 2018). NRG4 acts on ErbB3 and/or ErbB4 to induce β-oxidation and inhibit *de novo* lipogenesis in liver (Chen et al., 2017). Insulin acts via the insulin receptor to increase glucose uptake in all metabolic tissues while suppressing gluconeogenesis and inducing lipogenesis in the liver (Saltiel and Kahn, 2001; Samuel and Shulman, 2016; Vecchio et al., 2018). PDGF-AA acts through PDGFR-α and/or PDGFR-β to suppress hepatocyte insulin sensitivity (Abderrahmani et al., 2018), while PDGF-BB decreases insulin sensitivity in both the liver and white adipose tissue (Raines et al., 2011; Onogi et al., 2017). SCF promotes Pgc1α transcription and mitochondrial biogenesis in brown fat (Huang et al., 2014). CSF1 acts on CSF1R and induces lipid droplet gene expression, lipid accumulation, and increases hepatic Kupffer cells in the liver (Gow et al., 2014; Pridans et al., 2018). FGF1 acts on FGFR1 in the brain to suppress food intake (Suh et al., 2014; Scarlett et al., 2016). FGF5 acts on FGFR1 to suppress lipid accumulation in the liver (Hanaka et al., 2014). FGF10 acts on FGFR2 to increase adipogenesis in adipocytes (Sakaue et al., 2002; Asaki et al., 2004). FGF19 binds to β-Klotho/FGFR1/4 to induce β-oxidation, increase hepatic glycogen and protein synthesis, reduce lipogenesis in white adipose tissue; suppress food intake and improve glucose tolerance through actions in the brain (Tomlinson et al., 2002; Fu et al., 2004; Marcelin et al., 2014; Perry et al., 2015). FGF21 binds to FGFR1/β-Klotho to induce fatty acid (FA) oxidation, decrease triglycerides and improve insulin sensitivity in liver. FGF21 also increases glucose uptake, energy expenditure and improves insulin sensitivity by acting on muscle and adipose tissue. FGF21 inhibits food intake through central effects (Kharitonenkov et al., 2005; Coskun et al., 2008; Xu et al., 2009; Ge et al., 2011; Fisher et al., 2012; Bookout et al., 2013; Minard et al., 2016; BonDurant et al., 2017). HGF activates MET which induces glucose uptake in both adipocytes and myotubes (Bertola et al., 2007; Perdomo et al., 2008) decreases lipid accumulation in liver (Kosone et al., 2007), and increases glycogen synthesis and glucose uptake in hepatocytes (Fafalios et al., 2011). MSP binds to RON to inhibit lipid accumulation in the liver (Stuart et al., 2015; Chanda et al., 2016). GAS6 activates TAM receptor family members to decrease β-oxidation and increase inflammation in the liver (Fourcot et al., 2011). GDF15 acts on RET/GFRAL to induce mitochondrial respiration, lipolysis, and β-oxidation in both the liver and in adipose tissue (Chung et al., 2017). GDF15 also acts on the brain to suppress appetite (Tsai et al., 2013, 2014; Hsu et al., 2017; Yang et al., 2017; Patel et al., 2019).

Here we review emerging roles of RTK signaling in metabolic regulation and highlight unexpected roles for receptor families in regulating energy homeostasis, focusing on glucose and lipid metabolism (Figure 1 and Table 1). The metabolic functions of RTKs and their ligands, as well as the molecular mechanisms by which RTKs control metabolic processes will be discussed in the context of dysregulation of energy homeostasis and metabolic diseases including obesity, diabetes and ectopic lipid accumulation in the liver.

**TABLE 1:** Diverse functions of RTKs and their ligands in regulating metabolism.

Receptor	Target	Phenotype	References
**EGFR**			
EGFR	EGF protein treatment	Both increased insulin secretion, both decreased and increased glucose levels reported	Jansen et al., 2006; Lee et al., 2008
	Kinase dead EGFR	Decreased glucose levels	Li et al., 2018
	EGFR inhibitor Erlotinib	Decreased blood glucose levels, improved glucose tolerance and insulin sensitivity	Li et al., 2018
	EGFR inhibitor PD153035	Improved NAFLD and glucose tolerance	Choung et al., 2019
	Constitutively active EGFR	Increased plasma LDL cholesterol and triglycerides	Scheving et al., 2014
ErbB3/4	NRG1 protein treatment	Improved glucose tolerance and insulin sensitivity, lowered blood glucose, reduced weight gain	Ennequin et al., 2014; Ennequin et al., 2015; Zhang et al., 2018
	Nrg4 global KO	More prone to develop diet-induced insulin resistance and hepatic steatosis, fibrosis and inflammation	Wang. G.-X. et al., 2014; Guo et al., 2017
	NRG4 OE in adipose tissue	Improved diet-induced NASH	Guo et al., 2017
**PDGFR**			
PDGFR	PDGFββ protein treatment	No change in blood glucose after protein treatment	Yuasa et al., 2004
	Partial loss of PDGFβ activity	Decreased glucose levels, improved insulin sensitivity, decreased insulin levels	Raines et al., 2011
	PDGFRβ inducible global KO	Increased energy expenditure, alleviated diet-induced obesity, improved glucose metabolism	Onogi et al., 2017
	PDGFR/Kit inhibitor Imatinib	Decreased blood glucose, increased insulin sensitivity and glucose disposal, elevated adiponectin levels, decreased cholesterol-induced atherosclerosis	Breccia et al., 2004; Veneri et al., 2005; Agerkvist et al., 2008; Fitter et al., 2010; Gómez-sámano et al., 2018; Pouwer et al., 2018
Kit	Kit heterozygotes	Increased glucose levels, impaired glucose tolerance	Krishnamurthy et al., 2007
	Kit mutation	Impaired glucose tolerance and insulin sensitivity, increased body mass and body fat, increased serum triglyceride levels, decreased energy expenditure	Huang et al., 2014
	SCF global OE	Reduced weight gain, increased thermogenesis	Huang et al., 2014
CSF1R	CSF1-Fc protein treatment	Increased body weight	Gow et al., 2014
	CSF1-Fc protein treatment	No change in body weight	Pridans et al., 2018
**FGFR**			
FGFR1	FGF1 protein treatment	Decreased glucose levels without causing hypoglycemia, improved insulin sensitivity	Suh et al., 2014; Scarlett et al., 2016
	FGF1 KO	Impared glucose tolerance and insulin sensitivity	Jonker et al., 2012
	FGF5 global KO	Developed fatty liver	Hanaka et al., 2014
	FGF21 protein treatment	Decreased body weight, fat mass, glucose, lipid and insulin levels, increased	Kharitonenkov et al., 2005; Coskun et al., 2008; Xu et al., 2009
	FGF21 KO	Suppressed browning of white adipose tissue	Fisher et al., 2012
	FGF21 liver-specific KO	Impaired glucose tolerance and insulin sensitivity	Markan et al., 2014
	FGF21 adipocyte-specific KO	No change in glucose tolerance and insulin sensitivity	Markan et al., 2014
	FGFR1 adipose specific KO	Loss of FGF21-mediated lowering of glucose, insulin and triglycerides levels, body weight, and increase in energy expenditure; preserved functions of FGF19 treatments on decrease of glucose, insulin levels, and body weight	Adams et al., 2012b; Foltz et al., 2012
FGFR1/FGFR4	FGF19 protein treatment	Decreased body weight, glucose and insulin levels, increase energy expenditure and glucose tolerance, decrease HPA axis activity, increased blood lipid levels	Tomlinson et al., 2002; Fu et al., 2004; Morton et al., 2013; Wu et al., 2013; Perry et al., 2015
	FGF15 KO	Impaired insulin sensitivity and elevated serum cholesterol. decreased liver fibrosis under high-fat diet	Schumacher et al., 2017
	FGFR4 KO	Increased fat mass, increased circulating lipid levels	Huang et al., 2007
	FGFR inhibitor PD173074	Impaired glucose tolerance, increased food intake, increased plasma levels of norepinephrine and epinephrine	Ryan et al., 2013; Rojas et al., 2015
FGFR2	FGF10 KO adipocytes	Impaired adipocyte differentiation	Sakaue et al., 2002; Asaki et al., 2004
	βKlotho adipose specific KO	Loss of FGF21-mediated acute glucose lowering effect but preserved functions of long term treatment of both FGF19 and FGF21 on lowering body weight, glucose and insulin levels, and hepatic triglycerides	Adams et al., 2012a; Ding et al., 2012; BonDurant et al., 2017; Lan et al., 2017
HGFR MET	βKlotho whole-body KO	Loss of FGF21-mediated increase in energy expenditure and lowering of body weight, fat mass, and insulin levels	Ding et al., 2012
	HGF global OE	Ameliorated fatty liver, increased serum triglyceride levels	Kosone et al., 2007
	HGF OE in muscle	Improved glucose tolerance under high-fat diet	Sanchez-encinales et al., 2015
	HGF OE in heart	Protected from high-fat diet induced body weight gain, improved insulin sensitivity	Muratsu et al., 2017
	HGF antibody	Impaired glucose clearance	Muratsu et al., 2017
	MET knockdown in liver	Impaired glucose clearance	Fafalios et al., 2011
	MET KO in hepatocytes	No influence on liver lipid accumulation	Bhushan et al., 2019
RON	MSP global KO	Induced hepatic steatosis under normal diet, no effect on body weight	Bezerra et al., 1998
	RON global KO	Impaired glucose tolerance, protected from diet-induced obesity and liver steatosis	Stuart et al., 2015; Yu et al., 2016
**TAM**			
AXL	AXL OE in myeloid cells	Increased glucose and insulin levels, increased diet-induced body weight gain	Augustine et al., 1999
	AXL inhibitor R428	Reduced diet-induced body weight gain, reduced subcutaneous and gonadal fat mass	Lijnen and Christiaens, 2011
	AXL global KO	No effect on body weight or fat mass	Scroyen et al., 2012
	GAS6 global KO	Gained less diet-induced fat mass, protected from hepatic steatosis and fibrosis	Maquoi et al., 2005; Fourcot et al., 2011
**RET**			
RET/GFRAL	GDF15 global OE	Decreased body and fat mass, improved glucose clearance, decreased insulin levels	Baek et al., 2006; Macia et al., 2012; Chrysovergis et al., 2014; Wang. X. et al., 2014
	GDF15 global KO	Increased body weight, fat mass and food intake, more prone to develop NAFLD	Tsai et al., 2013; Kim et al., 2018
	GDF15 protein treatment	Decreased food intake and body weight, improved insulin sensitivity, taste aversion	Xiong et al., 2017; Chung et al., 2017; Patel et al., 2019

*Control of metabolism by RTKs and their ligands by using loss-or gain-of function models or by pharmacology. KO, knockout; OE, overexpression; LDL, low-density lipoprotein; NAFLD, non-alcoholic fatty liver disease; NASH, non-alcoholic steatohepatitis; HPA axis, hypothalamic pituitary adrenal axis.*

## EGF Receptor Family

Epidermal growth factor receptors (EGFR)/ERBB are a family of RTKs with essential roles in regulating cell proliferation, survival, differentiation and tissue development (Schneider and Wolf, 2009). The ErbB receptor family comprises four members: ErbB1 (epidermal growth factor receptor, EGFR), ErbB2 (EGFR2), ErbB3 (EGFR3), and ErbB4 (EGFR4), all of them being ubiquitously expressed in tissues of mesenchymal, epithelial and neuronal origin such as liver, muscle, adipose tissue and brain (Yano et al., 2003). EGFRs are activated by eleven cognate ligands, including epidermal growth factor (EGF), transforming growth factor-α (TGF-α), amphiregulin (AR), betacellulin (BTC), the ectodomain shedded heparin-binding EGF-like growth factor (HB-EGF), epiregulin (EPR), epigen (EPG), and neuregulins 1-4 (NRG 1-4) (Macdonald-Obermann and Pike, 2014). EGF can only activate EGFR, while NRG1 and NRG2 are ligands for both ErbB3 and ErbB4 (Jones et al., 1999). No ligand has been identified for ErbB2, but the receptor is a dimerization partner for other EGFRs. ErbB3 lacks tyrosine kinase activity but can still be phosphorylated at its tyrosine residue through interaction with the other EGFRs and transduce signals in response to ligand binding (Guy et al., 1994). The fact that EGFR ligands exhibit different receptor affinities and receptor binding specificities explains how such a large set of ligands can control distinct cellular functions. Other factors that contribute to the diversity of biological functions are tissue-specific receptor expression, ligand concentrations and ligand secretion. The complexities of the EGFR-ligand interactions, the ligand signaling redundancies and the functional selectivity for the EGFRs in physiology have been discussed extensively elsewhere (Wieduwilt and Moasser, 2008; Wilson et al., 2009; Macdonald-Obermann and Pike, 2014).

### Epidermal Growth Factor

The overall genetic and pharmacological studies of EGF-EGFR signaling in the context of metabolic regulation is controversial, but most studies indicate that inhibition of EGFR signaling improves insulin sensitivity. The 6 kDa secreted growth factor EGF share many biological activities with insulin, including the action on a tyrosine kinase receptor, inducing fatty acid and glycogen synthesis in rat hepatocytes and 3T3 fibroblasts, and stimulating glycolysis in cancer cells (Diamond et al., 1978; Bosch et al., 1986; Kaplan et al., 1990; Baulida et al., 1992). Notably, circulating EGF levels in diabetic leptin receptor-deficient (db/db) mice and streptozotocin-induced diabetic mice are reduced to approximately 30% of wild-type control mice (Kasayama et al., 1989). Based on these early studies and the fact that EGF receptors are present on pancreatic β-cells, EGF was initially investigated as a growth factor secreted from the excretory cells of the pancreas proposed to act as a regulator of glucose metabolism. The pancreas normally responds to high levels of glucose by inducing a rapid secretion of insulin which is a highly regulated and dynamic process (Rosenfeld, 2002). Studies using recombinant EGF treatment demonstrate that EGF also increases insulin secretion in mouse insulinoma cells and pancreatic islets at 50% of the levels obtained with glucose (Lee et al., 2008). The mechanism by which EGF regulates insulin secretion was demonstrated to involve the L-type Ca^2+^ channel influx, which is the canonical pathway downstream of insulin action. At least in cells, this effect could be fully reversed with pharmacological EGFR inhibition, suggesting that the effects of EGF on insulin secretion require activation through its receptor (Lee et al., 2008). Furthermore, administration of EGF to healthy and diabetic mice increases insulin levels and lowers glucose levels, suggesting that EGF acts in a feed-forward mechanism to increase insulin secretion. In contrast to these mouse studies, studies in rats demonstrated only a modest increase in plasma glucose levels after recombinant EGF administration (Jansen et al., 2006). Since the studies by Jansen et al. (2006) were not performed under clamped glucose conditions, it is unclear if the effect of EGF is secondary due to increased insulin or glucagon levels *in vivo*.

However, in contrast to the stimulatory effect of EGF administration on insulin secretion, pharmacological inhibition of EGFR activity using erlotinib lowers basal blood glucose levels, increased glucose tolerance and insulin sensitivity in mice (Li et al., 2018), but the mechanism for this effect is still unclear as EGFR is expressed ubiquitously. While the direct effect of EGF on controlling glucose uptake in peripheral tissues has never been tested *in vivo*, recombinant EGF treatment was shown to facilitate glucose transport in 3T3-L1 adipocytes overexpressing EGFR (Van Epps-Fung et al., 1996). The receptor expression of EGFR is downregulated in white adipose tissue from insulin-resistant and type 2 diabetic women (Rogers et al., 2012). In rodents, hepatocytes express a large number of EGFRs: each hepatocyte of an adult rodent liver expresses about 600,000 EGFRs (Scheving et al., 2014). Interestingly, the hepatic EGFR expression decreases during fasting (Feuers et al., 1989) and in type 1 and type 2 diabetes (Chua et al., 1991). EGFR is hyperphosphorylated in livers from high-fat diet-induced NAFLD mice, suggesting an activation of the EGFR signaling pathway. Inhibition of EGFR using PD153035 significantly improves the pathological signs of non-alcoholic fatty liver disease (NAFLD), including glucose tolerance, pAKT signaling and expression of *sterol responsive elementary binding protein (srebp) 1* and *2*, key transcriptional regulators of *de novo* lipogenesis. The suppression of lipogenesis and enhanced fatty acid oxidation results in a reduction in hepatic steatosis and hypercholesterolemia in mice (Choung et al., 2019). On the other hand, activation of the EGFR pathway in the Dsk5 mutant mice which harbor a mutation in the EGFR gene resulting in a ligand-independent, constitutively active receptor, leads to elevated liver cholesterol levels, liver enlargement, as well as increased plasma low-density lipoprotein (LDL) secretion and plasma triglycerides (Scheving et al., 2014). Therefore, it is plausible that EGF has direct and indirect effects on regulating both insulin secretion, glucose uptake and lipogenesis in all the peripheral organs. These somewhat opposing findings on EGF-EGFR activation in the context of glucose and insulin sensitivity raises the question about tissue-selective effects of EGFR signaling. Additional information gained from tissue-specific EGFR-KO mice and small molecule EGFR inhibitors can potentially shed light on the metabolic effects associated with perturbing this receptor pathway.

### The Neuregulins

A growing body of work demonstrates a metabolic role for the secreted neuregulins 1 and 4 (NRG1 and NRG4), a sub-family of EGF-like ligands structurally related to EGF that were originally discovered in the early 1990s. NRG1 is essential for the development of the nervous system and heart. Thus, global deletion of NRG1 results in embryonic lethality, while knockouts of NRG2-4 develop normally (Meyer and Birchmeier, 1995; Britto et al., 2004; Wang. G.-X. et al., 2014; Hayes et al., 2016). More recently, several lines of evidence point to a role of NRGs in the control of metabolism via actions on muscle, liver, adipose tissue and the hypothalamus (Wang. G.-X. et al., 2014; Zhang et al., 2018). The four *NRG* genes encode for several isoforms, most of which contain a transmembrane domain and an N-terminal extracellular EGF-like domain (Burden and Yarden, 1997). The soluble NRGs are generated from proteolytic cleavage of a transmembrane pro-NRG at the cell surface, releasing a smaller bioactive ligand containing the EGF-like domain that is sufficient for receptor binding and inducing a biological response. All four NRGs act mainly on ErbB3 and ErbB4 with no direct activity on EGFR and ErbB2, but they recruit ErbB1 and ErbB2 as co-receptors (Harari et al., 1999; Jones et al., 1999).

NRG1 was first identified as a 44 kDa glycoprotein in ras-transformed cells that could indirectly phosphorylate ErbB2 by binding ErbB3 and ErbB4 (Peles et al., 1992). Interestingly, the proteolytic cleavage of NRG1 is regulated by muscle contraction, suggesting a temporal regulation of NRG1 ligand secretion by changes in physical activity (Lebrasseur et al., 2003). This observation led to the hypothesis that neuregulin action may mimic certain aspects of exercise. Skeletal muscle adapts to acute and chronic exercise by controlling gene transcription and protein synthesis responsible for muscle remodeling, as well as glucose disposal and insulin sensitivity (Rabol et al., 2011; Deshmukh, 2016; Samuel and Shulman, 2016). For example, treatment with nanomolar levels of a recombinant NRG1 isoform, heregulin-beta1, acutely stimulates glucose uptake in muscle cells and skeletal muscle tissue to levels comparable to insulin, while chronic treatment increases mitochondrial oxidative function and insulin sensitivity (Suárez et al., 2001; Cantó et al., 2004; Canto et al., 2007). This effect is due to a translocation of the glucose transporters GLUT1, GLUT3, and GLUT4 in skeletal muscle. These glucose transporters are normally downregulated during differentiation of myogenic cells, an effect that can be reversed by the addition of NRG1. This suggests that NRG1 is involved in not only maintaining skeletal muscle function but can also directly regulate myogenesis during development or after skeletal muscle injury. Consistent with the myogenic actions on skeletal muscle cells, NRG1 is currently extensively explored as a growth factor involved in cardiac muscle regeneration in model organisms and human pilot clinical trials (Santoro and Sahara, 2015).

In addition to its role in muscle, NRG1 has also been shown to be important for whole-body glucose regulation by actions on liver, adipose tissue and brain. Several important studies have increased our understanding of how NRG1 exerts its metabolic effects through both peripheral and central actions. In leptin-receptor deficient diabetic db/db mice, acute and chronic treatment of NRG1 at a dose of 50 μg/kg improves glucose tolerance and lowers blood glucose, at least in part by suppressing hepatic glucose production (Ennequin et al., 2015). Based on receptor phosphorylation profiling, the action of NRG1 on suppression of hepatic gluconeogenesis is mediated by ErbB3 at Y1289, which triggers AKT activation and phosphorylation of FOXO1 in the liver (Ennequin et al., 2015; Zhang et al., 2018). Simultaneously, NRG1 also causes a massive increase in circulating leptin levels accompanied by a decrease in food intake. Although the mechanism for the suppression of food intake has not formally been demonstrated, the effect on food intake was lost in the db/db mice, suggesting that the central effects of NRG1 treatment is mediated by leptin (Ennequin et al., 2014). Moreover, treatment with a long-lived NRG1-Fc fusion protein containing the EGF-like domain also potently lowers blood glucose, increases insulin sensitivity, and promotes weight loss in mice fed a high-fat diet (Zhang et al., 2018). The central actions of NRG1 seem to be mediated by ErbB4 by the direct action on POMC neuron depolarization. The multiple pleiotropic actions of NRG1 warrant further mechanistic studies of NRG1 regulation, secretion and receptor activation in hypothalamus under basal, obese and diabetic conditions, and how they contribute to whole-body energy regulation. In addition to the direct effects on liver and brain, NRG1 can also induce the secretion of FGF21, although the effects on blood glucose, body weights and food intake by NRG1 are independent of FGF21 (Zhang et al., 2018). However, it is possible that other aspects of NRG1 signaling and activity are mediated by FGF21. Future work should focus on addressing the detailed mechanisms of regulation of glucose control, feeding behavior and hepatic glucose production upon ErbB3 and ErbB4 activation by NRG1 and other ligands.

The fourth neuregulin, NRG4, was recently found as a brown fat secreted factor controlling energy homeostasis (Wang. G.-X. et al., 2014). Brown fat serves an important function in the defense against cold environments by increasing the body temperature through the dissipation of chemical energy in the form of heat (Cohen and Spiegelman, 2015). Endocrine control of energy balance mediated by factors secreted from highly metabolic organs might provide important insights into the biology of energy regulation. Indeed, brown fat is a potent endocrine organ known to release factors such as FGF21, bone morphogenic proteins (BMPs) and interleukin-6 (IL-6) that control systemic metabolic functions (Villarroya et al., 2017). In addition to brown adipose tissue, nrg4 is also expressed in murine white adipose tissue and liver (Wang. G.-X. et al., 2014). Serum NRG4 concentrations are inversely associated with non-alcoholic fatty liver disease, metabolic syndrome and cardiovascular disease in obese humans, while elevated NRG4 is associated with a decreased risk of NAFLD in both children and adults (Cai et al., 2016; Jiang et al., 2016; Wang et al., 2019). In obese subjects, NRG4 expression is downregulated in white adipose tissue in human and both white and brown adipose tissue in mice, indicating that the expression is regulated by the abundance of nutrients or by hormone status, and raises the possibility that the severity of obesity is exacerbated by nrg4 deficiency. Consistent with this hypothesis, mice with a global deletion of nrg4 are more prone to develop diet-induced insulin resistance and hepatic steatosis, while fat-specific overexpression of nrg4 protects mice from obesity-associated metabolic dysfunction (Wang. G.-X. et al., 2014). Likewise, overexpression of nrg4 in adipose tissue protected mice from diet-induced NASH (Guo et al., 2017). Notably, this leads to a suppression of the nuclear form of *srebp1c*, as well as suppressing *srebp1c* target genes. Mechanistically, nrg4 transgenic expression lowers *de novo* lipogenesis and enhances fatty acid β-oxidation by activating both ErbB3 and ErbB4 receptors in the liver (Chen et al., 2017). However, these overexpression studies may not entirely recapitulate the signaling mechanisms and receptor binding preferences under physiological ligand concentrations. While the study by Chen et al. demonstrated evidence for a liver-selective binding of NRG4, it is also plausible that NRG4 may contribute to the regulation of whole-body metabolism by activating the receptors in other tissues.

In summary, studies over the last decade has brought a better understanding of the molecular mechanisms of energy regulation through the EGFR family. However, there are still many remaining questions. Several ligands of ErbB, including NRG1, NRG4, and EGF, have been shown to regulate glucose and lipid metabolism. Notably, one genome-wide association study identified two SNPs in the *NRG3* gene that are associated with basal metabolic rate and body mass index. However, the potential role of NRG3 in metabolism has never been formally demonstrated (Lee et al., 2016). Notably, the expression of ErbB3 itself increases during fasting and diabetes, a phenomenon that can be reversed by insulin administration, indicating that ErbB3 expression is under direct regulation of hormonal or nutrient status (Carver et al., 1997). In addition, a SNP at the *ErbB4* locus is strongly associated with higher BMI in an African American population (Salinas et al., 2016). Moreover, it is still unclear whether any of the other less characterized ligands Nrg2, TGF-α, AR, BTC, HB-EGF, EPR, or EPG are involved in regulating any aspects of energy control. Moreover, the mitogenic effects of NRGs in hepatocytes are unclear, although NRGs have been reported to be mitogenic in cardiomyocytes and pancreatic cells (South et al., 2013; Gemberling et al., 2015). As the mitogenic actions of NRGs may cause unwanted side effects when used as therapeutic molecules, this biological property needs to be taken into consideration. In conclusion, the tissue specificity, mitogenicity and downstream physiological and molecular mechanisms through which the EGF receptor ligands exert their metabolic effects warrant further investigation to facilitate translation of these molecules into clinical applications.

## PDGF Receptor Family

The class III RTK family include PDGFR-α, PDGFR-β, CSF-1R (Ems), c-Kit, and FLT3. PDGFR-α plays important roles in tumor cell growth, angiogenesis and organogenesis of lung, skin, CNS, and the skeleton, while PDGFR-β is involved in early hematopoiesis and in mediating recruitment and proliferation of pericytes in the vascular beds essential for vasculogenesis (Betsholtz, 2004; Andrae et al., 2008). Consequently, the knockout mice of PDGFR-α, PDGFR-β, PDGF-A, and PDGF-B are lethal or have severe developmental defects (Leveen et al., 1994; Soriano, 1994; Fruttiger et al., 1999; Betsholtz, 2004). The most established PDGFR-mediated signaling cascades include SOS1, Ras and ERK kinase responsible for inducing the transcriptional and functional effects (Wu et al., 2008; Farooqi and Siddik, 2015). The cognate ligands PDGF-A, PDGF-B, PDGF-C, PDGF-D are serum-derived mitogens secreted as proteolytically processed forms in secretory vesicles from vascular endothelial cells, macrophages, and vascular smooth muscle cells and act as homo-or-heterodimers formed by dimerization of A, B, C, or D-polypeptide chains. While PDGF-CC and PDGF-DD are secreted as inactive ligands, PDGF-AA, PDGF-BB, or PDGF-AB transduce signals by binding to PDGFR-α and β homo-or heterodimer tyrosine kinase receptors at the cell surface.

### PDGF-A and PDGF-B

The overall correlative, genetic and pharmacological *in vivo* evidence strongly suggest that activation of the PDGF pathway causes tissue fibrosis and metabolic dysfunction. Both PDGFA overexpression and hypomethylation at a CpG site in *PDGFA* are associated with an increased risk of developing insulin resistance, type 2 diabetes, and steatohepatitis. In obese patients, increased liver PDGF-AA levels are positively associated with insulin resistance (Abderrahmani et al., 2018). In humans, PDGF-BB levels in urine are significantly increased in type 2 diabetic patients as compared to healthy individuals (Bessa et al., 2012). In addition, renal biopsies show overexpression of PDGF-BB in patients with diabetic nephropathy, suggesting that PDGF-BB might contribute to the development of fibrosis (Bessa et al., 2012). Mechanistically, PDGF-AA is thought to contribute to insulin resistance by suppressing the expression of the insulin receptor and insulin receptor substrate-1 (Abderrahmani et al., 2018). Consistent with these studies, hepatic insulin sensitivity can be restored by PDGF-AA-blocking antibodies and PDGF receptor inhibitors, supporting the notion that the increased PDGF-AA signaling contributes to insulin resistance (Abderrahmani et al., 2018). However, early studies using PDGF ligands in cell culture experiments indicated that PDGF-BB increased glucose uptake in adipocytes and other cell types. Like insulin, PDGF-BB stimulated glucose transport by activating GLUT4 translocation through the activation of PI 3-kinase and the serine-threonine protein kinase Akt/protein kinase B in CHO cells overexpressing PDGFR-β (Kamohara et al., 1995). Moreover, these effects were independent of insulin, the insulin receptor, and IRS proteins in 3T3-L1 adipocytes overexpressing PDGFR-β (Whiteman et al., 2003). These studies suggested that PDGF can enhance glucose transport *in vitro* when overexpressing PDGFR-β. However, this *in vitro* phenomenon did not translate in a physiological setting as mice administered with recombinant PDGF-BB showed no significant reductions in blood glucose unless PDGFR-β was overexpressed (Yuasa et al., 2004). Therefore, the contribution of PDGF receptor activation to the physiological regulation of peripheral glucose uptake is most likely negligible.

One well-established connection between the PDGFR family members and metabolism is the notion that adipocytes emerge from vascular, or “mural” cells expressing PDGFR-β that are present on a subset of cells in close proximity to blood vessels (Cinti et al., 1984; Nougues et al., 1993; Gupta et al., 2012). PDGFR-α and PDGFR-β are pre-adipocyte receptors that are dramatically downregulated during adipocyte differentiation. Activation of PDGFR-α in mice by mutating the kinase domain that increases the kinase activity inhibits the formation of mature adipocytes while favoring the formation of stromal fibroblasts, suggesting that PDGFR-α activation causes adipose tissue fibrosis (Hepler et al., 2017; Sun et al., 2017). In obesity, the expansion of white adipose tissue involves a reciprocal regulation of hypertrophy of established adipocytes and *de novo* differentiation to lipid-filled adipocytes from precursor fibroblast-like cells. Thus, expansion of white adipose tissue through hypertrophy leads to metabolic dysfunction, while adipocyte differentiation can improve diabetes, as exemplified by agonism of PPARγ, the master regulator of adipocyte differentiation (Tontonoz et al., 1994). In mice, visceral adipose tissue expands from tissue-resident PDGFRβ+ perivascular, or mural, progenitor cells during high-fat diet feeding (Gupta et al., 2012). In addition, subcutaneous beige adipocytes arise from smooth muscle progenitor cells expressing PDGFR-α and PDGFR-β (Jiang et al., 2014; Long et al., 2014). These studies highlight the complexities of adipogenesis and the expansion of white adipose tissue *in vivo* and the potential role of PDGFR-α/β in this process.

Consistent with PDGF-BB’s role in maintaining vascular functions, the deletion of the heparan-sulfate proteoglycan-binding motif in PDGF-B leads to reduced retention of PDGF-BB in the matrix, and subsequently, preventing its main function as an angiogenic growth factor. When bred into the leptin-deficient ob/ob background, these PDGF-B retention-deficient mice demonstrate enhanced whole-body glucose homeostasis, increased insulin sensitivity and a reduction in circulating insulin (Raines et al., 2011). This provides evidence that increased vascular permeability through suppression of PDGF-B activity can improve diabetes in mice, although the mechanisms for this function are not fully understood. Similarly, inducible global PDGFR-β-KO mice display impaired pericyte detachment and reduced vascularity in eWAT, increased energy expenditure, lower body weights, as well as improved glucose metabolism (Onogi et al., 2017). Similarly, several studies in human cancer patients treated with inhibitors for class III RTKs have demonstrated glucose-lowering effects, which is consistent with an overall pathological function for the PDGFR pathway in metabolism. Imatinib (a dual PDGFR and c-Kit tyrosine kinase inhibitor) was originally approved for treating leukemia. Patients suffering from both diabetes and leukemia treated with imatinib demonstrated an unexpected lowering of blood glucose, and improvement of type 2 diabetes and elevated adiponectin levels (Breccia et al., 2004; Veneri et al., 2005; Fitter et al., 2010). Also diabetic individuals diagnosed with leukemia or gastrointestinal stromal tumors demonstrated a lowering of fasting plasma glucose and HbA1c after imatinib treatment (Gómez-sámano et al., 2018). Imatinib treatment also results in an improvement of insulin sensitivity and glucose disposal in insulin-resistant rats, but the mechanism behind this effect has not been established (Agerkvist et al., 2008). Additionally, treatment with imatinib in mice lowers cholesterol-induced atherosclerosis, although the mechanism for this effect is not clear (Pouwer et al., 2018). As PDGFR signaling inhibits adipogenesis, pharmacological blockade might enhance adipocyte differentiation in white adipose tissues (Fitter et al., 2012). If this hypothesis is true, this would provide a possible mechanism for improved glucose and lipid metabolism reported for a subset of imatinib-treated patients. These studies are limited by the use of multi-RTK inhibitors or by the phenotyping using global knockout mouse models of a receptor that is ubiquitously expressed. The lack of conditional knockout models in the study of the PDGF receptors in metabolism complicates the interpretation of which primary target tissue or cell type that is responsible for the phenotype. In addition, studies on ligand-receptor binding properties have demonstrated a large redundancy in the functional effects of PDGFR-α and PDGFR-β, making it difficult to distinguish the physiological roles and importance of the individual receptors (Wu et al., 2008).

In conclusion, a large body of evidence points toward a pathological contribution of activation of the PDGF receptors to metabolic dysfunction, including insulin resistance and liver fibrosis. Further studies elucidating the mechanisms by which PDGFR blockade improves insulin action in muscle and adipose tissue might increase our understanding of the role of this receptor family and open up potential opportunities for disease targeting.

### Stem Cell Factor (SCF)

The c-Kit receptor tyrosine kinase (CD117) is a stem cell growth factor receptor belonging to the PDGFR family and is expressed in a variety of tissues, including the pancreas (Li et al., 2019). Stem cell factor (SCF) is the only known ligand for c-Kit and is produced by fibroblasts, stromal cells, keratinocytes, endothelial cells, and is critical for growth of multiple lineages of progenitor cells. The human gene *KITLG* (mouse *kitlg*) encodes two alternatively spliced isoforms generating a ~30 kDa transmembrane protein and a protein with a proteolytic site in exon 6 allowing for release as a soluble ~20 kDa protein that can be detected in human serum averaging 3 ng/ml (Flanagan et al., 1991; Langley et al., 1993). The two isoforms display different expression patterns and appear to have distinct abilities to transmit signals, at least *in vitro*. Both the soluble and long membrane-bound proteins of SCF form homodimers, and the dimerization appears important for bioactivity. Activation with the soluble isoform results in rapid and transient c-Kit phosphorylation, while stimulation of cells using the membrane-associated form results in persistent and prolonged activation of the receptor as well as the downstream MAPK pathway (Miyazawa et al., 1995). The differences in cellular responses might be explained by the inability of the membrane-associated form to be internalized. Although most *in vitro* studies and plasma measurements have been conducted using the soluble form and will be here referred to as soluble CSF, recognizing this dual ligand action is important in particular when studying the complete loss of function models of CSF.

The role of c-Kit in fetal rat and human endocrine pancreatic development, survival, and function has been well characterized (Yashpal et al., 2004; Krishnamurthy et al., 2007; Li et al., 2019). Soluble SCF acts on the pancreas, adipose tissue and muscle by inducing PI3-K and JAK/STAT signaling to stimulate proliferation, migration and survival (Ronnstrand, 2004). Interestingly, expression levels of *kitl* and the *c-Kit* receptor are regulated by energy overload in mice and in humans. *c-Kit* expression is significantly downregulated in both white and brown adipose tissue from diet-induced obese mice, while serum levels of SCF are elevated in both diet and genetically (db/db) induced obese and diabetic mice, which is suggestive of SCF resistance. Consistent with the secretion profile in mice, serum levels of soluble SCF in humans positively correlates with BMI (Huang et al., 2014). Global c-Kit deficient mice and the Steel-Dickie (spontaneous SCF-KO) mice develop severe anemia (Krishnamurthy et al., 2007). However, mice that are heterozygotes for c-Kit have been studied in the context of glucose regulation. Loss of one *c-Kit* allele leads to high fasting blood glucose levels and impaired glucose tolerance, mainly due to compromised insulin secretion *in vivo*. Moreover, β-cell mass was significantly reduced in c-Kit heterozygotes compared with controls, suggesting that c-Kit might regulate β-cell function (Krishnamurthy et al., 2007). Interestingly, mice with a missense mutation in the c-Kit gene as a result of chemical mutagenesis have impaired tyrosine kinase activity and develop obesity and peripheral insulin resistance. This phenotype was suggested to be due to decreased heat production, energy expenditure and mitochondrial dysfunction in brown adipose tissue and skeletal muscle. Conversely, increasing the levels of SCF in serum by overexpressing the soluble form of SCF increases thermogenesis and reduces weight gain, potentially by promoting *Ppargc1a* (PGC-1α) transcription and mitochondrial biogenesis (Huang et al., 2014). However, it is questionable if the reported 10% induction of circulating SCF in this study is sufficient to drive increased thermogenesis and weight loss in other organisms. Direct evidence of weight loss effects and increased thermogenesis should be performed by pharmacological means using recombinant CSF administration in the presence or absence of c-Kit inhibitors. Overall, these data provide some mechanistic insight that increasing SCF/c-Kit signaling can improve energy homeostasis by both enhancing β-cell function and energy expenditure in brown adipose tissue and skeletal muscle, but more studies are needed to confirm these findings. Another study reported that c-Kit mice with a chemical-induced hypomorphic point mutation displayed increased juvenile hepatic steatosis, suggesting that c-Kit also can control aspects of lipid metabolism in the liver (Magnol et al., 2007). The PI3K/AKT and JAK/STAT pathways are known regulators of hepatic lipid metabolism, but the mechanism for c-Kit-dependent regulation of metabolism via these pathways remains to be determined. In addition, these phenotypes may be attributed to strain-specific mutations as chemical-induced point mutations might affect the expression of neighboring genes. The many observed peripheral metabolic functions of c-Kit and SCF are surprising given the fact that c-Kit is mainly expressed in the developing embryo as well as in hematopoietic lineage cells and immune cells, while the expression of c-Kit in adult metabolic organs is very low, indicating that the effects might be indirect. Further investigations into the regulation and function of SCF/c-Kit signaling using tissue-specific knockout models will be an intriguing direction for future research.

### Colony-Stimulating Factor 1 (CSF1)

Colony-stimulating factor 1 (CSF1)/Macrophage colony-stimulating factor 1 (M-CSF) is the ligand for CSF1R and is produced by multiple cell types, including fibroblasts, bone marrow stromal cells, brain astrocytes and endothelial cells. Signals initiated by CSF1R control survival, differentiation, and proliferation of cells of the mononuclear phagocyte lineage by regulating the secretion of proinflammatory chemokines (Hume and Macdonald, 2012; Jenkins and Hume, 2014; Chitu et al., 2017). The *CSF1* gene encodes for a transmembrane protein which upon proteolytic cleavage releases the active form of CSF1 with a predicted molecular mass of 26 kDa as a monomer. CSF1 has established pleiotropic roles in postnatal somatic growth. The studies of CSF1 in energy metabolism are limited, but emerging evidence suggests that CSF1 is a homeostatic regulator of hepatic lipid metabolism by acting on immune cells or hepatocytes in the liver. In mice, CSF1 treatment suppresses several transcriptional insulin targets genes in the liver, including genes encoding enzymes involved in gluconeogenesis, fatty acid oxidation, and amino acid catabolism (Gow et al., 2014). Systemic administration of a CSF1-Fc fusion protein with prolonged pharmacokinetic properties demonstrated a 50% increase in proliferation of liver and spleen, which can be compared to a 15–37% increase when mice are treated with the mitogenic hepatocyte growth factor (HGF) (Gow et al., 2014). CSF1 also expands the macrophage populations in blood and organs, including hepatic Kupffer cells in the liver. The increase in liver size and in Kupffer cell count was also replicated in another study when administering recombinant CSF1 to neonatal rats (Pridans et al., 2018). In addition, neonatal exposure to CSF1 leads to elevated hepatic lipid accumulation and increased expression of genes involved in lipid droplet formation, a phenotype that was never examined in the adult mice (Pridans et al., 2018). CSF1 also plays a role in lipoprotein clearance by reducing polyunsaturated esters, suggesting a plausible mechanism whereby CSF1 can promote the recruitment of immune cells responsible for atherosclerotic plaque formation (de Villiers et al., 1994; Jessup et al., 1997). However, it is still unclear whether the actions of CSF1 are pro-or anti-atherogenic and the mechanisms by which CSF1 contribute to cholesterol levels and lipid metabolism will need to be further elucidated. As CSF1R is expressed in hepatic immune cells as well as in hepatocytes, further analysis of the functional outcomes of CSF1 action on the particular cell types in the liver would be of interest. Moreover, inconsistent effects on body weight have been reported, with either a body weight gain in adults or no effects on body weight in neonatal rats after recombinant CSF1 treatment, which may imply that CSF1 has different functions at specific developmental stages (Gow et al., 2014; Pridans et al., 2018). Overall, these findings indicate distinct actions of CSF1 depending on the developmental stage, which warrants further age-dependent and cell-type specific studies in the context of metabolic disease, especially hepatic steatosis and atherosclerosis.

## FGF Receptor Family

The functional roles of FGFs in physiology and disease are well established. Activation of fibroblast growth factor receptors (FGFR) induces cellular responses controlling growth, proliferation, differentiation, and survival (Tiong et al., 2013). The FGF family consists of 22 members of paracrine, endocrine or intracrine FGFs. The paracrine FGFs are FGF1-10 and FGF16-18, while the endocrine FGFs are FGF19 (and its rodent ortholog FGF15), FGF21, and FGF23 (Gasser et al., 2017). The FGF homologous factors FGF11-FGF14 do not activate FGFRs and are not therefore generally considered members of the FGF family. Over the past decades, extensive and impressive studies of FGFR-mediated control of glucose and lipid metabolism through the unique ligands FGF1, FGF19, and FGF21 have dramatically increased our understanding of the powerful and diverse metabolic actions of these pathways (Kharitonenkov and Adams, 2014; Markan and Potthoff, 2016; Gasser et al., 2017; Maratos-Flier, 2017; Somm and Jornayvaz, 2018; Tezze et al., 2019). The biology of FGF21 has been comprehensively reviewed elsewhere (Kharitonenkov and DiMarchi, 2015). The discussion here will focus on the biological diversity and the similarities between the FGF ligands in the regulation of metabolism.

### FGF1

Emerging data revealing striking effects of the paracrine FGF1 on glucose homeostasis have renewed the interest of this growth factor that was discovered many years ago (Burgess and Maciag, 1989). The global FGF1-KO mouse is viable and normal without any apparent developmental phenotype (Miller et al., 2000). FGF1 is ubiquitously expressed, elevated in white adipose tissues of ob/ob mice and high-fat diet fed mice (Jonker et al., 2012; Choi et al., 2016; Gasser et al., 2017). Studies using human samples have reported both a positive correlation of FGF1 serum levels with insulin resistance (Wang et al., 2018) as well as inverse correlations of FGF1 with BMI and blood triglycerides (Zhu et al., 2017). Whether this regulation is age or hormone-dependent is worthy of further investigation. Interestingly, Evans and colleagues discovered that FGF1 is a transcriptional target of PPARγ and is therefore increased during adipocyte differentiation (Jonker et al., 2012). When the FGF1-KO mice are challenged with a high-fat diet for 16 weeks, they develop severe glucose intolerance and insulin resistance. Consistent with the loss of function studies, both peripheral and central pharmacological administration of FGF1 recombinant protein show dramatic glucose-lowering effects without causing hypoglycemia (Suh et al., 2014; Scarlett et al., 2016). A single peripheral injection of FGF1 in diabetic rodents normalizes diabetes within hours, while multiple doses promote insulin sensitization in as short as 3 weeks (Suh et al., 2014). In comparison, a single intracerebroventricular (i.c.v.) injection of FGF1 lowers circulating glucose in about a week and this effect is sustained beyond 16 weeks without insulin sensitization. This suggests potential differences in the mechanisms of FGF1 action depending on the target tissue, which may include secondary effects on insulin sensitivity. Notably, the anti-diabetic effect is not secondary to weight loss, and the mechanisms and specific neuronal circuits by which FGF1 induce diabetes remission remains to be fully determined but likely involves the HPA axis (Perry et al., 2015). Unlike FGF21, both FGF19 and FGF1 are classical mitogens. Therefore, a partial agonist was developed by mutating the heparin-binding domains in FGF1, which demonstrated abolished proliferative capacity while maintaining the metabolic effects (Huang et al., 2017). These engineering approaches could open up new areas of biology in addition to developing new therapeutic applications.

### FGF21 and FGF19

The systemic glucose and lipid regulatory functions and mechanisms of FGF19 and FGF21 have been extensively studied during the past decades. However, the relative importance of the central nervous system, adipose tissue, and liver in the long-term metabolic actions of these FGF family members are still intensely studied. FGF19 binds FGFR1 and uniquely binds FGFR4. In contrast, FGFR1 appears to be the preferred receptor for FGF21 (Adams et al., 2012b; Foltz et al., 2012). While FGF1 can bind to FGFR directly, the binding of FGF21 and FGF19 to FGFRs requires the scaffolding protein β-Klotho as a co-receptor to elicit cellular signaling (Adams et al., 2012a; Ding et al., 2012; Kharitonenkov and DiMarchi, 2015). Therefore, although the FGFRs are expressed in multiple tissues, the distinct β-Klotho expression pattern in the brain, liver, adipose tissue and pancreas, in combination with the preferred FGFR determines the target organs of the endocrine FGFs (Markan and Potthoff, 2016).

Both FGF19 and FGF21 are postprandial hormones that regulate metabolic processes in particular during fasting and feeding. While FGF21 is increased during fasting in rodent models, plasma levels of FGF21 are increased both after acute high energy intake and after prolonged fasting in humans (Gillum, 2018). FGF21 levels are also increased in type 2 diabetes and is positively correlated with BMI, insulin resistance, hyperglycemia, NAFLD, hyperlipidemia and hepatic triglycerides (Zhang et al., 2008; Li et al., 2010; Chen et al., 2011). Therefore, elevated FGF21 levels may be a predictor for metabolic syndrome and type 2 diabetes (Fisher et al., 2010). The increased levels of FGF21 in metabolic disorders are suggestive of FGF21 resistance, similar to that of insulin and leptin. However, FGF21 is inactivated by proteolytical cleavage in plasma. The fact that immunochemical kits for the plasma level determination of FGF21 cannot distinguish between active and cleaved, inactive FGF21 suggests that the increased circulating levels of FGF21 may not reflect true FGF21 resistance which warrants further biochemical studies.

Many metabolic functions are shared between FGF21 and FGF19. They both reduce body weight, glucose and insulin levels, and cause an increase in energy expenditure in obese rodent models (Tomlinson et al., 2002; Fu et al., 2004; Kharitonenkov et al., 2005; Coskun et al., 2008; Xu et al., 2009). They also activate sympathetic outflow to BAT and induce changes in thermogenic gene expression (Owen et al., 2014; Douris et al., 2015; Lan et al., 2017). Both FGF19 and FGF21 reduce liver triglyceride levels, increase insulin sensitivity and stimulate whole-body glucose uptake by acting directly on adipose tissue (Fisher et al., 2011; Kir et al., 2011b; Lan et al., 2017).

FGF21 and FGF19 exert their metabolic effects via overlapping and distinct receptors in peripheral organs and in the central nervous system (Fu et al., 2004; Sarruf et al., 2010; Kir et al., 2011a; Morton et al., 2013; Ryan et al., 2013; Marcelin et al., 2014; Perry et al., 2015; Samms et al., 2017). Detailed mechanistic studies have demonstrated that FGF21 activates MAPK and the downstream effectors ERK1/ERK2 and induces the expression of GLUT1 as well as mTORC1/S6K, resulting in increased glucose uptake in adipocytes (Moyers et al., 2007; Ge et al., 2011; Minard et al., 2016). Elegant studies of the βKlotho-KO and FGFR-KO mice demonstrate that the action of FGF21 is eliminated the absence of the receptor and co-receptor (Adams et al., 2012a; Foltz et al., 2012). A recent study also shows that the acute, but not chronic, glucose-lowering effects of FGF21 depends on FGF21 signaling to brown adipose tissue (BonDurant et al., 2017). Moreover, FGF21 induces browning of white adipose tissue accompanied by increases in adipose PGC-1α and *ucp1* expression. Similarly, mice with a global FGF21 ablation display an impaired ability to adapt to chronic cold exposure, with suppressed browning of white adipose tissue (Fisher et al., 2012). While FGF21 is mainly considered a hepatokine, it has also been reported to be released from skeletal muscle (Kharitonenkov and DiMarchi, 2015). Studies performed in skeletal muscle have reported that FGF21 induces glucose uptake by increasing GLUT1 expression and enhancing GLUT1 abundance at the plasma membrane without changes in AKT or AMPK phosphorylation (Mashili et al., 2011). The translation of this result to humans is questionable because no significant β-Klotho expression has been detected in human skeletal muscle (Petryszak et al., 2016).

On the other hand, some unique actions of FGF19 are mediated by FGF19-FGFR4 binding on hepatocytes, including the suppression of bile acids via downregulation of the rate-limiting enzyme for bile acid synthesis, *cyp7a1* (Wu et al., 2011). Human *FGF19* is expressed in the liver and gallbladder while the expression of its mouse ortholog *fgf15* is restricted to the distal part of the intestine in mice (Somm and Jornayvaz, 2018). In contrast to FGF21, FGF19 levels are reduced in obese humans and the circulating FGF19 levels are negatively correlated with BMI, circulating triglycerides and HDL cholesterol (Barutcuoglu et al., 2011; Hu et al., 2018). Similarly, FGF19 levels are reduced in obese adolescents with NAFLD (Wojcik et al., 2012). In the liver, FGF19 stimulates protein and glycogen synthesis through FGFR4 mediated ERK-RSK signaling, which suppresses GSK3, increases glycogen synthase activity, and enhances glycogen storage (Kir et al., 2011a). FGF19 has also been shown to reduce *acetyl-CoA 2 carboxylase* expression in the liver, which leads to an increase in lipid oxidation and a decrease of hepatic triglyceride levels (Tomlinson et al., 2002; Fu et al., 2004). A recent study shows that FGF19 improves hepatic steatosis by promoting HDL biogenesis and cholesterol efflux from the liver by activation of LXR (Zhou et al., 2019). Global FGFR4-KO mice have increased fat mass, circulating lipid levels and are insulin resistant. Surprisingly, while restoration of FGFR4 selectively in hepatocytes in the FGFR4-KO mice normalizes plasma lipid levels, it fails to restore the glucose intolerance and insulin resistance (Huang et al., 2007). However, FGF19 treatment still improves glucose tolerance in FGFR4-KO mice, indicating that the activation of FGFR4 is not essential for the systemic glucose regulation function of FGF19 (Wu et al., 2011). Moreover, fgf15-KO mice develop insulin resistance and elevated serum cholesterol, but demonstrate improved liver fibrosis (Schumacher et al., 2017). This may be a result of selective activation of FGFR1 and FGFR4 in hepatocytes and non-parenchymal cells. Overall, these studies suggest additional roles of FGF19 and FGFR4 in other organs or cell types.

In hepatocytes, FGF19 suppresses fatty acid synthesis through inhibiting the expression of *srebp-1c*, which is accompanied by inhibition of lipogenic enzyme expression (Bhatnagar et al., 2009). It is, however, unclear if the regulation of lipid synthesis by FGF19 in liver contributes to the circulating lipid levels. Interestingly, a recent study shows that the actions of FGF19 and FGF21 in liver and adipose tissue are not required for their longer-term effects on weight loss and glycemic control, while β-Klotho expression in neurons is essential for both weight loss, glucose-lowering and regulation of insulin levels by FGF19 and FGF21 (Lan et al., 2017). FGF19 can suppress the HPA axis and AGRP/NPY neuronal activity to control eating behavior and energy homeostasis (Marcelin et al., 2014; Perry et al., 2015). Central administration of FGF19 also improves glucose tolerance (Morton et al., 2013). The metabolic effects of FGFR signaling in the CNS using genetic models or protein treatments are largely recapitulated using selective FGFR inhibitors. I.c.v. administration using the FGFR inhibitor PD173074 causes glucose intolerance in healthy mice (Ryan et al., 2013; Rojas et al., 2015). In addition, PD173074 pre-treatment blunted the glucose-lowering effect of systemic FGF19 treatment when administered i.c.v., suggesting that the effect of FGF19 is mainly via central action (Morton et al., 2013).

Recent advances illuminating novel FGF biology by a subset of ligands, mainly FGF1, FGF19, and FGF21, have opened up a new area of research in energy homeostasis by this diverse protein family. However, the potential involvement of other FGFs in energy homeostasis is less understood. Recently, two artificial short peptides developed based on the paracrine FGF8 and FGF17 sequences can improve glucose homeostasis after 4 days injections although the mechanism, receptors and target tissues were not elucidated (Liu et al., 2018). The third endocrine FGF, FGF23 has been shown to be involved in minerals and vitamin D metabolism (Hu et al., 2013). A correlational study showed that besides BMI, FGF23 levels are also correlated with HOMA-IR (Fayed et al., 2018). However, other studies demonstrated no vitamin D-independent functions of FGF23 in glucose homeostasis, insulin signaling or fat metabolism in mice (Streicher et al., 2012). Finally, the paracrine FGF5 and FGF10 have been shown to regulate lipid accumulation in liver, and adipogenesis in fat, respectively (Sakaue et al., 2002; Asaki et al., 2004; Hanaka et al., 2014). Further studies are needed to evaluate the role of the other FGFs in metabolism and their underlying mechanisms of action.

## HGF Receptor Family

The hepatocyte growth factor receptor (HGFR) family includes the receptors MET and RON and their ligands HGF and MSP, respectively. MET is widely expressed in epithelial cells in many tissue types including the liver and pancreas, prostate, kidney, muscle and bone marrow (Stuart et al., 2000; Comoglio et al., 2008). The ligand for MET, hepatocyte growth factor (HGF) was first identified as a soluble mitogen for hepatocytes promoting growth and liver regeneration in 1989, but HGF is also expressed in skeletal muscle, adipose tissue, as well as in pancreatic β-cells (Nakamura et al., 1989; Rahimi et al., 1994; Zarnegar and Michalopoulos, 1995; Otonkoski et al., 1996; Fain et al., 2004; Bell et al., 2006). HGF is synthesized as a full length pre-pro-HGF with an N-terminal signal peptide for classical secretion (Baldanzi and Graziani, 2014). HGF undergoes proteolytic cleavage by several serum proteases to generate the biologically active HGF molecule that consists of a heterodimer of a 34 kDa light β chain and a 69 kDa heavy α chain linked with a disulfide bond (Nakamura et al., 1987). MET is essential for numerous cellular functions, including mitogenesis, angiogenesis, and anti-apoptosis. Hence, HGF and MET are indispensable for development, as evidenced by the embryonic lethality seen in the global knockout mice (Bladt et al., 1995; Schmidt et al., 1995). Numerous studies of tissue-specific knockout mice have demonstrated the importance of this pathway during development and in maintaining tissue homeostasis, and have been extensively reviewed elsewhere (Kato, 2017).

### Hepatocyte Growth Factor (HGF)

A large body of literature strongly suggests that activation of the HGF-MET pathway improves glucose tolerance and reduces lipid accumulation. Interestingly, the HGF-MET pathway seems to be tightly regulated by nutritional status. In three independent studies, circulating HGF levels have been shown to be elevated in obesity, diabetes and metabolic syndrome (Rehman et al., 2003; Hiratsuka et al., 2005; Rajpathak et al., 2010). Moreover, HGF in circulation is positively correlated with the mass of perivascular fat, waist circumference, body mass index, body fat content and the development of insulin resistance (Vistoropsky et al., 2009; Rittig et al., 2012; Courten et al., 2013; Tsukagawa et al., 2013; Bancks et al., 2016).

The first pharmacological studies using HGF were performed in rats. Repeated administration of human recombinant HGF demonstrated prevented liver fibrosis (Matsuda et al., 1997). The same group later reported that 7 days of HGF treatment at a dose of 200 μg/kg also could reverse alcohol-induced fatty liver by enhancing lipid secretion from hepatocytes (Tahara et al., 1999). In isolated rat hepatocytes, HGF transiently inhibits the release of lipids (triacylglycerol, total cholesterol, and phospholipids) in 12 h but stimulates their release at 36 h (Kaibori et al., 1998). In HepG2 cells, HGF treatment also reduces the intracellular lipid content by stimulating the expression of microsomal triglyceride transfer protein and apolipoprotein B (Kosone et al., 2007). These studies are supported by a whole-body overexpression mouse model of HGF, which is protected from high-fat diet-induced fatty liver. Consequently, these mice demonstrate reduced lipid accumulation and activation of microsomal triglyceride transfer protein and apolipoprotein B (Kosone et al., 2007). Unexpectedly, hepatocyte-specific MET deletion did not induce fatty liver development in mice fed fast-food diet for 5 months (Bhushan et al., 2019). This suggests that the control of lipid synthesis and secretion by HGF might be mediated by other cell types than hepatocytes.

Besides the function in liver, transgenic mice with muscle-specific overexpression of HGF displays an improved systemic glucose tolerance under high-fat diet. These mice also exhibits an increase in AKT phosphorylation levels in the gastrocnemius muscles (Sanchez-encinales et al., 2015). In addition, cardiac-specific overexpression of HGF resulting in a fourfold increase in circulating HGF levels is sufficient to protect mice from high-fat diet-induced body weight gain and insulin resistance. These mice also demonstrate reduced accumulation of macrophages and reduced levels of inflammatory factors in white adipose tissue compared to wild-type mice (Muratsu et al., 2017). Consistent with the improved glucose tolerance when overexpressed, a HGF-neutralizing antibody in wild-type mice exacerbated the symptoms of diet-induced obesity and impaired glucose clearance ability, but the tissues responsible for this phenotype were not identified (Muratsu et al., 2017). Mechanistically, activation of the HGF/MET pathway increases glucose uptake in peripheral metabolic organs and stimulates insulin secretion in pancreatic β-cells. In 3T3-L1 adipocytes, HGF increases glucose uptake by promoting GLUT4 translocation and the activation of PI3K (Bertola et al., 2007). In skeletal muscle myotubes, HGF also increases glucose transport and plasma membrane expression of GLUT-1 and GLUT-4 mediated by the PI3K/AKT pathway (Perdomo et al., 2008). HGF also stimulates glucose uptake and glycogen synthesis in both human and rodent primary hepatocytes (Fafalios et al., 2011). The precise molecular mechanisms by which HGF regulates lipid metabolism have still to be determined.

Intriguingly, similar to the insulin receptor (IR), MET is an αβ heterodimer held together by disulfide bonds. This structural and sequence similarity to the IR led to the interesting discovery that Met engages the insulin receptor in a Met-IR hybrid complex to regulate the cellular insulin response by interacting with and phosphorylating the IR (Fafalios et al., 2011). Injection of insulin into mice expressing an albumin promoter-driven dominant negative MET receptor results in hyperglycemia, reduced insulin sensitivity and glucose clearance, suggesting that a MET is required for a normal insulin response by the liver. The IR-MET crosstalk appears to be restricted to the liver where both receptors are highly expressed, as no cooperation was seen in white adipose tissue and skeletal muscle (Fafalios et al., 2011). While gluconeogenesis was suppressed upon HGF protein treatment in this study, other important downstream target pathways of insulin in the liver, such as lipogenesis, were not assessed. Considering the earlier studies on HGF in controlling lipid synthesis in the liver (Kaibori et al., 1998), it would be of priority to determine to what extent this effect is mediated by the action of IR-MET. The studies by Fafalios et al. (2011) are also limited by the report of only of the insulin receptor B isoforms. It would be informative to determine whether the MET crosstalk occurs with other insulin receptor isoforms or with the IGF-Rs. Additional studies are needed to clarify the functional role and the biological significance of the IR-MET crosstalk in liver metabolism.

Overall, the existing literature using gain- and loss- of-function studies demonstrate a protective role of the HGF/MET pathway in obesity and insulin resistance by acting on several organs including the liver and skeletal muscle. While HGF can reduce lipid accumulation in the liver, the downstream mechanisms of this function remain to be determined, and whether MET activation in other cell types can indirectly control liver lipid metabolism is unknown. In addition, it is unclear if the HGF/MET levels and signaling pathways are regulated by nutrient status in the liver, muscle and adipose tissues and the underlying mechanisms of regulation. The major concern regarding the activation of HGF/MET pathway for therapeutic purposes *in vivo* is the mitogenic effects that might lead to increased tumor growth. The MET kinase inhibitor SU11274 has been studied in non-small cell lung cancer xenografts resulting in inhibition of tumor growth (Tang et al., 2008), but whether pharmacological administration of HGF can lead to cancer development in the absence of oncogene or tumor suppressor alterations is unclear. It is also possible that the mitogenic properties of HGF could be uncoupled from the metabolic effects, as previously shown to be possible with FGF1 (Muratsu et al., 2017). In addition, the metabolic effects of highly selective MET inhibitors such as capmatinib in regulating glucose or lipid metabolism have not been shown in either animal models or in humans. These insights are key to our understanding of the functional and physiological roles of HGF/MET signaling in metabolic disease.

### Macrophage-Stimulating Protein (MSP)

MSP (also known as hepatocyte growth factor-like, HGFL) was originally isolated as a bioactive fraction from plasma that could activate peritoneal macrophages (Skeel et al., 1991), but the *MSP* expression is also high in hepatocytes (Chen et al., 1997). MSP is a 78 kDa disulfide-linked heterodimer that shares considerable homology with HGF and is involved in regulating proliferation, cell migration and cell shape (Chen et al., 1997). Ron, also called Macrophage-stimulating protein receptor (MST1R), is a member of the MET protooncogene family and is activated by MSP (Gaudino et al., 1994). *Ron* is expressed in tissue-resident macrophages and cells of epithelial origin such as colon, breast, and skin (Wang et al., 2006).

Studies of MSP/RON signaling in physiology are limited, but some evidence points toward a beneficial role for MSP/Ron signaling in both glucose and lipid metabolism in the liver, both by direct action on hepatocytes and also on tissue-resident macrophages (Kupffer cells). The entire Ron gene deletion results in embryonic lethality. However, the global Ron receptor knockout mice where the ligand-binding domain is deleted develop severe obesity and glucose intolerance under high-fat diet (Yu et al., 2016). On the other hand, studies on Ron knockout mice lacking the tyrosine kinase domain rendering the protein inactive demonstrated the opposing finding that ablation of Ron signaling protected the mice from high-fat diet induced obesity and hepatic steatosis (Stuart et al., 2015). The reason for this discrepancy is unclear but might be related to the strain differences (FVB versus C57BL/6) or to the method of gene targeting in these mice. MSP whole-body knockout mice develop hepatic steatosis under chow diet without apparent effects on body weights (Bezerra et al., 1998). Mechanistically, *in vitro* studies in primary rat hepatocytes have shown that MSP acts through the AMPK pathway to suppress the expression of *pepck and glc-6-pase* and thus reduce hepatic gluconeogenesis (Chanda et al., 2009). In primary mouse hepatocytes, in addition to the activation of the AMPK pathway, MSP treatment also inhibits lipotoxicity gene expression induced by lipopolysaccharide and palmitic acid. Similar effects were found in HepG2 cells, where MSP protected against palmitic acid-induced lipogenic gene expression and lipid accumulation (Chanda et al., 2016). Lastly, in an attempt to mimic NASH *ex vivo*, bone marrow-derived macrophages were challenged with oxidized low-density lipoprotein and LPS, which inhibits AMPK activity and increases inflammation. Treatment with MSP protein restored AMPK activity and suppressed pro-inflammatory cytokine gene expression and secretion (Chanda et al., 2016). However, another study that investigated the functions of MSP in the early stage of NASH using the LDLR knockout mice was not able to confirm the effects of MSP in ameliorating NASH. In fact, MSP-treated mice showed increased gene expression of pro-inflammatory and pro-apoptotic mediators in the liver (Li et al., 2016). Given the role of Ron in regulating innate immune responses (Wilson et al., 2008), further studies on liver inflammation in the context of NASH are warranted. These interesting but opposing observations indicate that MSP may suppress glucose production and lipid accumulation in the liver, but more mechanistic studies are needed to better understand the role of Ron signaling in the development of fatty liver disease. Additionally, no correlation between metabolic disease such as NASH and the levels of Ron or MSP expression in mice or humans have been demonstrated. Detailed mechanistic understanding of the mechanisms of regulating lipid accumulation will be important to fully understand the role of MSP in physiology.

Intriguingly, Ron has been shown to physically interact and crosstalk with other RTKs such as Met, PDGFR, EGFR and the insulin receptor family, highlighting the complex regulation and signaling complexities in biology (Follenzi et al., 2000; Peace et al., 2003; Kobayashi et al., 2009; Potratz et al., 2010; Jaquish et al., 2011). Therefore, the interpretation of the physiology in the Ron KO mice might be confounded by this crosstalk such that the Ron-RTK interaction may contribute to whole-body regulation of physiology even in the absence or presence of the Ron tyrosine kinase domain or the ligand-binding domain. Determining the direct and indirect effects of MSP on Ron signaling and the importance of the RTK crosstalk is essential to elucidate the biological and physiological effects.

## TAM Receptor Family

The TAM receptors Axl, Mertk and Tyro3 were originally found in the nervous system, but later shown to be ubiquitously expressed (Lai and Lemke, 1991). The identification of their ligands protein S and GAS6 in 1995 have further revealed their pleiotropic functions in cell growth, proliferation, apoptosis, coagulation and inflammation (Nakano et al., 1995; Stitt et al., 1995; Varnum et al., 1995; Goruppi et al., 1996; Nagata et al., 1996; van der Meer et al., 2014). GAS6 is expressed in adipose tissue, heart, kidney, lung, and liver and binds to the receptor tyrosine kinase Axl with a 100–1000-fold higher affinity over Tyro3 and Mertk. The activation of TAM downstream signaling pathways such as PI3K, ERK and NF-kB leads to pro-inflammatory cytokine production and platelet aggregation. While both Protein S and GAS6 mediate coagulation and wound healing, GAS6 is identified as the only ligand that can control systemic metabolism.

The role of Axl in metabolism was initially identified in 1999 when studying transgenic mice overexpressing *Axl* under a myeloid promoter. As Axl receptors are highly expressed in myeloid cells, these mice were generated to study its function in the progression of leukemia (Augustine et al., 1999). Unexpectedly, these mice were obese, diabetic and insulin resistant. A study from Lijnen and Christiaens (2011) later found that inhibition of Axl using the selective Axl inhibitor R428 reduced weight gain in mice fed a high-fat diet. The whole-body GAS6-KO mice also have reduced subcutaneous and gonadal fat mass when fed a high-fat diet (Maquoi et al., 2005). As the mouse phenotypes were not due to behavioral changes in food intake or in activity levels, it led to the hypothesis that GAS6-Axl activation may directly impair glucose and lipid metabolism in peripheral organs. However, the global Axl-KO mice did not show any difference in body weight gain or subcutaneous and gonadal fat mass under either standard or high-fat diet (Scroyen et al., 2012). This could be due to compensatory effects by the other two TAM receptors that were upregulated in the Axl-KO mice (Scroyen et al., 2012). *In vitro*, GAS6 directly induces proliferation of preadipocytes and promotes preadipocytes differentiation into mature adipocytes (Maquoi et al., 2005), while Axl inhibitor R428 suppresses 3T3-F442A differentiation (Lijnen and Christiaens, 2011). The expression levels of GAS6 and all TAM receptors increases during differentiation of embryonic stem cells but decreases during 3T3-F442A differentiation (Lijnen and Christiaens, 2011). This effect is likely related to the expression pattern of TAM receptors. While Gas6, Mertk, and Tyro3 are expressed in mature murine adipocytes, the Axl expression is more restricted to pre-adipocytes. The physiological role of GAS6 in adipose tissue is still unclear as no adipocyte-specific GAS6-KO mouse has been generated. Considering the relatively low Axl receptor expression in adipose tissue, it is unclear whether activation of this pathway in adipose tissue has any physiological importance.

The predominant mechanism explaining the metabolic phenotypes are still unknown, but the prevailing hypothesis is that activation of the Axl pathway leads to chronic inflammation and fibrosis, mainly in white adipose tissue and the liver. First, Axl overexpression under the myeloid promoter results in increased circulating levels of TNF-α (Augustine et al., 1999). TNF-α activates inflammation pathways that can induce insulin resistance in adipose tissue (Hotamisligil et al., 1993) and other metabolic organs. Second, activation of Axl in stellate cells induces the expression of the profibrotic genes *α-sma* and *col1a1*, which can be inhibited by the Axl inhibitor bemcentinib (Tutusaus et al., 2019). GAS6 and Axl are expressed in stellate cells and Kupffer cells, but not in hepatocytes (Couchie et al., 2005; Lafdil et al., 2006). Similarly, loss of GAS6 reduces recruitment of circulating monocytes and accumulation of myofibroblasts during liver injury, which leads to a suppression in liver inflammation and fibrosis (Lafdil et al., 2009; Smirne et al., 2019). GAS6-KO mice also display reduced liver fibrosis induced by chronic carbon tetrachloride treatment (Lafdil et al., 2009; Fourcot et al., 2011). Compared to wild-type mice, GAS6-KO mice have reduced liver mass, hepatic lipid accumulation and inflammation induced by a diet deficient in choline. These effects were accompanied by increased β-oxidation indicated by gene expression as well as downregulation of genes involved in inflammation such as *il-1β, tnf-α*, and *tnf-c*. Surprisingly, a recent study showed that GAS6 activation via Mertk in hepatocytes protects from cell death induced by palmitic acid lipotoxicity, but is pro-fibrogenic in stellate cells and pro-inflammatory in Kupffer cells where the Axl receptor is more highly expressed (Tutusaus et al., 2019). The functions of Tyro3 activation in different liver cell types were not tested in this study. It is unclear whether the liver phenotype is a primary effect of hepatic GAS6 deficiency or is secondary due to global metabolic changes. *In vivo* experiments testing whether direct targeting of Axl, Mertk or Tyro3 by using specific TAM inhibitors or by GAS6 neutralizing antibodies can reduce the development of hepatic fibrosis are still underway. There are significant roadblocks that limit our understanding of the biology of GAS6/TAM signaling. First, while GAS6 can bind all the TAM receptors, whether the three members have distinct functions or activate distinct downstream pathways are not fully understood. Tissue-specific deletions of GAS6 and the TAM receptors will enable the elucidation of tissue-specific functions of GAS6 as well as determine the unique functions of each type of TAM receptor. Although mice lacking any single or two receptors are viable and fertile, mice with a deficiency of all three receptors are infertile and develop autoimmune disease (Lu and Lemke, 2001). The crosstalk and compensatory mechanisms between Axl, Mertk, and Tyro3 need to be further investigated to determine the necessity of developing a receptor-specific inhibitor. The three TAM members differ in expression patterns and functions, yet they share high structural homology, which is a challenge for developing small molecules with receptor specificity.

## RET Receptor Family

RET is ubiquitously expressed and is required for the development of the brain and of multiple peripheral organs (Tsuzuki et al., 1995). RET is also expressed in tumors and contributes to tumor progression (Mulligan, 2014). The whole-body RET knockout mice are not viable because of impaired brain and kidney development. Tissue-specific RET knockouts have been generated, but no metabolic effects have been reported under basal conditions (Kramer et al., 2007; Fonseca-Pereira et al., 2014; Ibiza et al., 2016). Remarkably, the ligands of RET all belong to the transforming growth factor-β (TGFβ) superfamily. In contrast to most other RTKs, RET does not bind to ligands directly, but only interact with ligands via obligate co-receptors. The canonical RET ligands are glial cell line-derived neurotrophic factors (GDNFs), including GDNF, neurturin (NTRN), artemin (ARTN), and persephin (PSPN). The activation of RET by GDNFs requires any of the co-receptor GDNF family receptor-α (GFRα) family members.

A recently identified RET ligand is GDF15, a member of the TGFβ superfamily that is expressed in immune cells and is upregulated in tissues in response to injury. GDF15 is a non-RTK cytokine that mediates an unusual signaling crosstalk between the receptor family species (Hsu et al., 2017). The interaction between GDF15 and RET requires the co-receptor GDNF family receptor α–like (GFRAL), which is a distant homolog of the GFRα family (Mullican et al., 2017; Emmerson et al., 2017). The expression of GFRAL is highest in the brain, especially hindbrain, and is weakly expressed in peripheral tissues (Mullican et al., 2017). The GDF15-GFRAL-RET interaction represents a mechanism of signal diversification and unique crosstalk across receptor families and exemplifies how immune cells can send signals to the brain under conditions of high metabolic stress.

The metabolic functions of GDF15 were first discovered when human GDF15 was overexpressed in mice to investigate its role in cancer progression. In addition to the suppression of colon carcinogenesis, interestingly, these mice lost weight and fat mass (Baek et al., 2006). Conversely, GDF15 knockout mice present with higher body weight, fat mass and food intake (Tsai et al., 2013). The function of GDF15 in mediating weight loss was later confirmed using overexpression of mouse GDF15 which lowered body weight and fat mass, a phenomenon that was accompanied by improved glucose clearance capacity and lowered circulating insulin levels (Macia et al., 2012; Chrysovergis et al., 2014; Wang. X. et al., 2014). Consistent with the genetic studies, pharmacological treatment with recombinant GDF15 robustly reduced food intake and body weight, which was accompanied by improved metabolic profiles in obese models of mice, rats, and monkeys (Xiong et al., 2017). In addition, recombinant GDF15 protein treatments for 3 weeks had dramatic effects on lowering body weights and improving insulin sensitivity in ob/ob mice (Chung et al., 2017). GDF15 is also a correlative biomarker for metabolic syndrome. For example, elevated circulating levels of GDF15 have been observed in mice, rats, and humans with obesity and NASH (Xiong et al., 2017; Kim et al., 2018).

The effects of GDF15 on whole-body metabolism have been proposed to be mediated by both central and peripheral effects. The mechanism by which GDF15 elicits its anorectic effects is through activation of the postrema (AP) and nucleus tractus solitarius area in the brainstem, which triggers downstream phosphorylation of ERK and AKT pathways. Neuronal activation by GDF15 has also been shown in the hypothalamus and amygdala (Tsai et al., 2013, 2014; Hsu et al., 2017; Yang et al., 2017). Interestingly, a recent study found that GDF15 treatment also induced other behavioral changes including a taste aversion of saccharin in mice (Patel et al., 2019). These observations can provide insights into how different brain regions crosstalk and how that is regulated by GDF15. The complete reversal of the actions of GDF15 in the GFRAL knockout animals together with the selective GFRAL brain expression strongly suggests that the main action is mediated by the brain. Other studies have suggested that the suppression of food intake may not explain all the beneficial effects of GDF15 on energy metabolism. Mice overexpressing GDF15 also demonstrate higher O_2_ consumption, CO_2_ production and heat generation that may contribute to the weight loss effects (Chrysovergis et al., 2014). In several studies, the changes in body mass and fat content were independent of any changes in food intake (Baek et al., 2006; Chrysovergis et al., 2014; Kim et al., 2018). In mouse models of diet-induced NASH, GDF15 deficiency exacerbated the steatosis, inflammation and fibrosis in the liver, but whether these effects are indirect or independent of body weight is still being debated (Kim et al., 2018). GDF15 has also been proposed to induce lipolysis and thermogenesis in brown and white adipose tissue, as higher expression of the lipolysis target genes *atgl*, *hsl* and the thermogenic gene *ucp1* has been observed after GDF15 treatment (Wang. X. et al., 2014). Also liver and skeletal muscle seem to respond to GDF15 with higher expression of lipolytic and oxidative phosphorylation genes following GDF15 administration (Chung et al., 2017). *In vitro*, GDF15 treatment induces phosphorylation of SMAD and ERK1/2 in differentiated 3T3-L1 adipocytes and primary mouse hepatocytes. These findings indicate that GDF15 may act on peripheral metabolic organs to increase lipolysis, oxidation, and thus increase whole-body energy expenditure. However, the peripheral metabolic functions of GDF15 need to be further investigated in models with tissue-specific ablations of RET, as the expression in peripheral tissues is relatively low (Kramer et al., 2007; Fonseca-Pereira et al., 2014; Ibiza et al., 2016). Ultimately, it remains to be determined whether GDF15 can suppress food intake and improve metabolic health in humans. It would also be of interest to investigate whether RET and GFRAL are regulated under nutrient deprivation and metabolic stress. Lastly, it would be valuable to know whether any of the canonical RET ligands GDNF, NTRN, ARTN, and PSPN are involved in the regulation of central or peripheral metabolism.

## Conclusion and Future Perspectives

Although an increasing number of drugs are available for appetite, glucose, and lipid control for obesity-related disorders (Hruby and Hu, 2015; Nathan, 2015), we still need a better understanding of the mechanisms, signaling mediators and pathways involved in systemic energy control, and how these pathways are dysregulated in obesity, type 2 diabetes and fatty liver. Increasing our understanding of the underlying mechanisms involved in systemic metabolic control by the identification of new ligand-receptor pairs could provide new insights and potentially new therapeutic targets.

Several family members belonging to the RTK family have recently been shown to control glucose and lipid metabolism, but the downstream mechanisms and the importance of activating these pathways in metabolism are still largely unknown. Notably, many RTK ligands or their receptors are regulated by physiological stimuli such as fasting, feeding, or nutrient overload, highlighting their roles as active sensors of organismal energy homeostasis. There is a growing appreciation for the signal diversification that occurs downstream of receptor tyrosine kinase activation. Explaining how a single ligand-receptor pair can exert such pleiotropic biological effects in a tissue-and context-dependent manner is still a remaining question. In addition, whether other families of RTKs are involved in regulating systemic metabolic control remains to be tested. Interestingly, the fascinating crosstalk between the RTK family and the TGFβ receptor family through GDF15 illustrates a more complex network of signals that control homeostatic regulation than previously appreciated. How can RTK signaling pathways crosstalk within and across receptor families, and how are these signals regulated under physiological and pathological stress Future studies should focus on identifying additional important ligands acting through these receptor complexes and their functions in regulating energy homeostasis.

## Author Contributions

MZ and KS designed the literature search and wrote the review. ZJ and YJ critically analyzed and revised the manuscript.

## Conflict of Interest

The authors declare that the research was conducted in the absence of any commercial or financial relationships that could be construed as a potential conflict of interest.
